# An Exposome Perspective on Environmental Enteric Dysfunction

**DOI:** 10.1289/ehp.1510459

**Published:** 2015-12-29

**Authors:** Job O. Mapesa, Amy L. Maxwell, Elizabeth P. Ryan

**Affiliations:** 1 Department of Environmental and Radiological Health Sciences, Colorado State University, Fort Collins, Colorado, USA; 2 Department of Public Health and Human Nutrition and Dietetics, Kenya Methodist University, Nairobi, Kenya; 3 Department of Health Sciences, University of Alaska Anchorage, Anchorage, Alaska, USA

## Abstract

**Background:**

Environmental exposures to chemicals have been shown to influence gastrointestinal function, yet little is known regarding whether chemical mixtures may be involved in the development of a subclinical enteric dysfunction found in infants and children born into poor hygiene and sanitation. Advances in gastrointestinal and immunotoxicology fields merit inclusion in complex discussions of environmental enteric dysfunction (EED) that severely affects children in developing countries.

**Objective:**

We aimed to highlight exposome approaches for investigating the potential influence of environmental chemical exposures on EED development, including a role for toxicant modulation of gut immune system and microbiome function.

**Discussion:**

A major focus on fecal–oral contamination in impoverished living conditions already exists for EED, and should now expand to include environmental chemicals such as pesticides and heavy metals that may be anthropogenic or dietary or from microbial sources. A comprehensive characterization of environmental chemical exposures prenatally and occurring in infants and young children will enhance our knowledge of any associated risks for EED and stunting.

**Conclusions:**

Integrating EED, chemical exposure, and stunting at various ages during childhood will enhance our apparent limited view when evaluating EED. Etiology and intervention studies should evaluate the suite of environmental chemical exposures as candidates in the composite of EED biomarkers.

**Citation:**

Mapesa JO, Maxwell AL, Ryan EP. 2016. An exposome perspective on environmental enteric dysfunction. Environ Health Perspect 124:1121–1126; http://dx.doi.org/10.1289/ehp.1510459

## Introduction

Environmental enteropathy (EE) and environmental enteric dysfunction (EED) are terms used to describe the same pathophysiological, subclinical condition of reduced small intestinal barrier and absorptive function that has high prevalence among children living in low- to middle-income countries where poor hygiene, inadequate sanitation, and malnutrition pervade (Crane et al. 2015; Keusch et al. 2013). The spectrum of EED involves structural and functional changes to the gastrointestinal tract (GI) that may include, but not be limited to, altered villous architecture, impaired mucosal immunity, nutrient malabsorption, and growth faltering (Lin et al. 2013; Lindenbaum et al. 1972). Chronic enteric pathogen exposures, including asymptomatic infections, and intestinal permeability in young children have thus far been central to EED research (Salazar-Lindo et al. 2004). However, we suggest that we explore the possible role for diversity of environmental toxicant exposures as well as dysbiosis of the gut microbiome from birth to 2 years of age to address major gaps in our knowledge of EED. The host burden and the host responses to toxicant exposures are highlighted in the concept of an “enteric dysfunction exposome” (Vrijheid et al. 2014). Across global geography and age groups, EED may be influenced in ways that have not yet been connected to existing knowledge of toxicologic importance, and this commentary highlights the compelling case for xenobiotics to be investigated in EED etiology. The enteric dysfunction exposome would not be limited to enteric pathogens and mycotoxins, but would encompass chemical classes for a wide range of environmental toxicants [i.e., endocrine disruptors, trace heavy metals, persistent organic pollutants (POPs), volatile organic chemicals (VOCs), and behaviors] (Miller and Jones 2014). The presence of chemical exposures in maternal blood and breast milk may affect infant immune tolerance, gut microbiome colonization, small intestinal development, and nutrient availability and absorption during *in utero*, prenatal, and postnatal periods; yet these combination of factors remain poorly characterized in EED-endemic regions (Crane et al. 2015; Gordon et al. 2012; Rappaport et al. 2014; Vrijheid et al. 2014). Thus, EED evaluation should include xenobiotic exposures that can be monitored noninvasively through blood, urine, saliva, and/or stool using both nontargeted omics-based and targeted measurements of exogenous and endogenous small molecules (Keusch et al. 2014). This approach exhibits strong potential to not only identify a suite of reliable EED exposure biomarkers but also design interventions that can perturb an EED-susceptible exposome (Rappaport 2011; Rappaport and Smith 2010; Vrijheid et al. 2014).

Studies in Bangladesh (Lin et al. 2013), Brazil (dos Reis et al. 2007), The Gambia (Campbell et al. 2003, 2004), Nepal (Langford et al. 2011), Malawi (Agapova et al. 2013; Galpin et al. 2005), and Tanzania (Mduma et al. 2014) demonstrate that EED is widespread and pervasive. The current list of EED-associated morbidities provides a strong rationale for identifying biomarkers, diagnostics, preventive agents, and sustainable treatment solutions (Keusch et al. 2014). Along with prevalent severe acute malnutrition– and undernutrition-related childhood mortalities, stunting is postulated to be secondary to EED (Keusch et al. 2013), with EED being a possible contributor to multiple generations of the 171 million children being affected by stunting globally (de Onis et al. 2013). In a case–control study of 202 stunted Zimbabwean infants, a measurement of the biomarkers of intestinal inflammation revealed that exposure to low-grade, chronic inflammation *in utero* and during early postnatal phases of life was associated with stunting that was likely due to extensive enteropathy that occurs during infancy (Prendergast et al. 2014). We suggest that the milieu of EED causative agents, epigenetic, and genetic factors merit elucidation in order to fill the gap in our knowledge regarding when and how EED can be controlled or prevented. Thus, we can hypothesize that multiple layers of environmental, microbiological, pharmacological, and dietary interventions are needed to reasonably reduce EED prevalence. Identification of EED biomarkers is the subject of ongoing global health research, and as such opens opportunities for new diagnostics and therapeutics (Mbuya and Humphrey 2016; Prendergast et al. 2015). The nature of EED research in children of developing countries merits inclusion of the spectrum of host–microbe interactions and chemical exposure diversity; investigating the combined effect could advance EED risk assessment, improve EED diagnostics and therapeutics, and deploy EED prevention initiatives.

## EED Characteristics, Etiology, and Epidemiology

**Figure 1:**
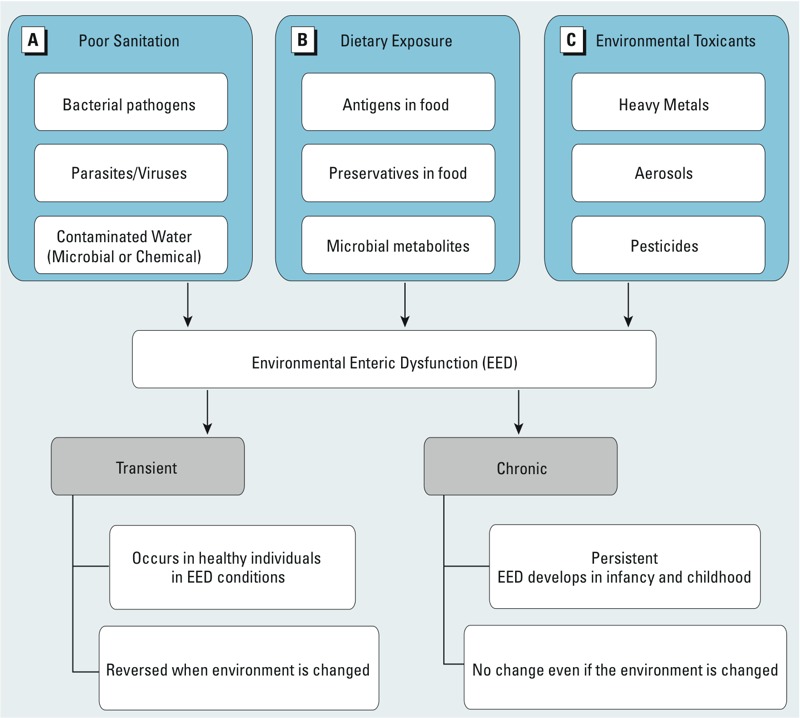
Environmental exposures that contribute to transient or chronic/persistent EED: (*A*) conventional factors thought to be responsible for EED; (*B*) dietary exposure factors known to influence the gut microbiota ecology; (*C*) environmental toxicants with potential to affect intestinal function and physiology. Dietary exposure and environmental toxicants are emerging factors that can be included in EED associated biomarker identification studies. This classification can help determine the drivers of transient or chronic EED states.

EED is a subclinical disorder characterized by abnormal morphology and physiology of the small bowel; specifically, it features increased gut permeability, altered gut villous architecture and function, nutrient malabsorption, and growth faltering (Lin et al. 2013; Lindenbaum et al. 1972; Prendergast and Kelly 2012). Gastrointestinal tissue biopsies of children with EED display crypt hyperplasia, villous atrophy, lymphocyte infiltration into the lamina propria and epithelium, and reduced mucosal surface area (Lin et al. 2013). The biopsies are also characterized by T-cell activation and heightened Th1 (T helper cell 1) cellular immune responses similar to what is seen in celiac sprue. This is not consistent with allergic responses, but rather appears to be a response to specific pathogens, presumably from ingestion of food and water containing fecal contaminants (Campbell et al. 2003). The intestinal epithelium creates a physical barrier between the external and internal environments in which the intracellular tight junctions and the apical brush borders prevent microbial attachment and invasion (Shen and Turner 2006). EED develops and occurs in the absence of overt manifestation of diarrhea; it was originally referred to as tropical enteropathy in the 1970s when a moderate number of documented cases of abnormal jejunal biopsies were identified from persons in tropical regions (Lindenbaum et al. 1972). Intestinal pathology varied geographically, and the condition resolved when affected individuals migrated to developed regions (Lindenbaum 1973). Subsequent studies in varied populations around the world showed that tropical enteropathy did in fact exist throughout the tropics, but that it was also absent in some tropical populations of high socioeconomic status, such as Qatar and Singapore. This illuminated the premise that environmental conditions were critical drivers of the condition, instead of geographical position (Prendergast and Kelly 2012). We believe that the unsanitary conditions in which EED-affected individuals reside contribute to the overriding causal factors of EED. However, we postulate that multiple sources of exposures contribute to both the transient and chronic/persistent nature of EED (Figure 1). In addition to poor sanitation, dietary contaminants and environmental toxicants can be equally hazardous or exert additive and or synergistic effects on the developing gastrointestinal tract via the microbiome, and thus merit qualitative and quantitative assessments for contributions to EED (Breton et al. 2013; De Filippo et al. 2010; Zhang et al. 2015). Although EED has been shown to be reversible if acquired in adulthood, as in the adult Peace Corps volunteers study (Lindenbaum et al. 1972), adults who resided in impoverished areas throughout their lifetimes demonstrate that EED acquired in childhood is chronic and difficult to reverse even after relocating to clean environments (Kelly et al. 2004; Keusch et al. 2013). For example, in a longitudinal cohort study of healthy African adults in Zambia, endoscopic biopsies of the proximal jejunem were obtained serially over 3 years to assess for changes in mucosal architecture in response to environmental conditions. At baseline and over the duration of the study, the entire cohort revealed the absence of predominant finger-like villi—an abnormal biopsy finding representative of EED—even though these adults were “healthy” and asymptomatic of intestinal infection (Kelly et al. 2004).

## Microbial and Dietary Origins of EED

Metagenomic studies of infant and childhood stool samples show that early postnatal environmental exposures have a pivotal role in shaping the predominant phylogenic structure of gut microbiota, and that this microbial configuration occurs rapidly during the first 2 years of life (Kau et al. 2011; Koenig et al. 2011). A recent Global Enteric Multicenter Study (GEMS) of diarrhea in young children in Mali, The Gambia, Kenya, and Bangladesh reported that during the first year of life, a healthy infant gut microbiota is characterized by a comparatively low diversity as well as a relatively high proportion of facultative anaerobes, and potentially pathogenic organisms that are believed to play a role in the development of host immune system. A high-throughput 16S rRNA gene sequencing was used to compare fecal microbiota composition in 992 children < 5 years of age who had been diagnosed with moderate to severe diarrhea with the microbiota from diarrhea-free controls subjects (Pop et al. 2014). Predominant bacteria vary among different populations of children, possibly in relation to diet (De Filippo et al. 2010; Pop et al. 2014; Wu et al. 2011; Yatsunenko et al. 2012). According to Subramanian et al. (2014), gut microbiota immaturity was defined by relative microbiota diversity and microbiota-for-age *z*-score indices. In addition to detecting limited changes in a group of malnourished children split into two dietary treatments, they reported reduced bacterial diversity with detection of 220 significantly different operational taxonomic units, 165 of which had diminished proportional representation in the stool microbiota of severely malnourished children compared with healthy children (Subramanian et al. 2014). Thus, the possible role for an immature gut microbiota associated with low-dose toxicant exposures in EED merits continued investigation (Hall et al. 2007; Subramanian et al. 2014) because the acquisition and composition of gut microbiota has also shown dependence on many factors including region of birth, history of hospitalization, mode of delivery, infant weaning, diet, age, sex, presence of siblings, infections, and antibiotic use (Miller and Jones 2014; Rappaport 2011; Vrijheid et al. 2014; Yatsunenko et al. 2012). It is plausible that EED may cause zinc deficiency by reducing its absorption, yet enteric infections can impair zinc homeostasis, increase deficiency, and aggravate EED by weakening gut barrier functions, which elevates incidences of GI tract infection and inflammation as a result of decreased gut absorptive capacity (Lindenmayer et al. 2014). Chronic parasitic infections with *Ascaris*, hookworms, and *Trichuris* may trigger or perpetuate EED via multiple inflammatory pathways (Bartelt et al. 2013).

Understanding EED as a congregation of changes in small-intestine function can help prevent malnutrition and stunting in infants in developing countries (Keusch et al. 2013). EED development has been associated with unrestrained enteric T-cell activation by persistent and abnormal concentrations of ingested fecal bacteria in the small intestinal lumen (Humphrey 2009). During nutrient processing, commensal bacteria secrete antimicrobial compounds that prevent infections by pathogenic microbes, and commensals support immune system functions to achieve homeostasis (Caricilli et al. 2014). It has been proposed that disruption of the gut homeostatic balance in children living in pathogen-laden environments supports low-grade chronic immune system stimulation that can culminate into small intestine–function impairment (Ngure et al. 2014). We propose that chemical toxicant exposures merit investigation alongside these pathogens as contributors to low-grade chronic immune stimulation.

## The Exposome

Applying an exposome lens to enhance the current understanding of EED may have advantages given the strong evidence already linking dietary exposures, malnutrition, immature gut microbiota, and diarrheal disease–causing pathogens (Salazar-Lindo et al. 2004). The exposome is defined as “the cumulative measure of environmental influences and associated biological responses throughout the lifespan, including exposures from the environment, diet, and behavior” (Miller and Jones 2014). The exposome includes relevant exposures from food, breast milk, water, air and soil, as well as microbes (e.g., bacteria, fungus, yeasts, archaea), toxicants, and food allergens (Lioy and Rappaport 2011). By evaluating the entire exposome in relation to EED, we may identify missing links in the multiple causative factors that contribute to EED. The exposome integrates overlapping domains of general and specific external factors along with the internal environment of the host (Caricilli et al. 2014). The exposome seeks to enumerate all of the possible sources of exposure and integrate biological data (Lioy and Rappaport 2011; Rappaport 2012), and would incorporate a more holistic picture of environmental exposures to EED epidemiological studies.

Chemical toxicants and gut microbiome studies have revealed meaningful interactions. For example, nonabsorbed heavy metals have a direct impact on the gut microbiota (Breton et al. 2013). We postulate that dietary exposure from breast milk, food, and water to multiple classes of pesticides might be a major contributor to EED. Children are uniquely sensitive to toxic chemicals in the environment, with greater concentrations of exposure to toxicants with respect to their body weight. More studies are needed to understand the interaction between the gut microbiome and xenobiotics in EED-prone regions given that xenobiotics affect physiology, metabolism, and gene expression of the human gut microbiome (Maurice et al. 2013).

Mycotoxins are common contaminants in foods such as maize, oats, rye, barley, wheat, and peanuts. An impaired intestinal integrity similar to EED has been demonstrated in animal model experiments following aflatoxin poisoning (Prendergast and Kelly 2012). Aflatoxin, fumonisin, and deoxynivalenol may have similar EED characteristics by which they impair the gut to induce stunting (Smith et al. 2012). These mycotoxins may also share a convergent pathway resulting in mucosal changes seen in EED (Smith et al. 2012). Specifically, it has been proposed that aflatoxins may affect child growth by collectively reducing zinc bioavailability, impairing protein synthesis and nutrient metabolism, and damaging the enterocytes. Sequential insults by these mycotoxins in combination with chemical toxicants may affect children at their most vulnerable developmental stages and serially interrupt critical developmental milestones (Bartelt et al. 2013).

The gut microbiome is central to growth and nutritional status through nutrient transformation as well as immune system and metabolic signaling (Jones et al. 2014). A diminished mucosal surface area and damage to the epithelium may impede nutrient absorption and lead to malnutrition. For instance, aberrations in the gut microbiome have been implicated as casual factors in Kwashiorkor, a form of severe acute malnutrition resulting from inadequate nutrient intake in addition to environmental factors (Smith et al. 2013). Early-life functional changes in the GI tract may herald and be exacerbated by a myriad of malnutrition drivers including inadequate diet, poverty, food insecurity, and infection with enteric pathogens, culminating in stunting (Keusch et al. 2014).

The microbiome produces many metabolites that greatly influence host response, as seen in vitamin and amino acid nutrient processing during infancy (Yatsunenko et al. 2012). Related faulty nutrient processing has been implicated in malnutrition, which subsequently increases susceptibility to infectious diseases (Dorrestein et al. 2014). Research shows that dietary habits shape nutrigenomics in an evolutionary fashion via influences from human genetics and gut microbiota (Daniell and Ryan 2012), and that systematic changes in dietary habits can lead to changes in the microbiota, functionally affecting the host nutrition status and immune responses (Kau et al. 2011). For instance, children from The Gambia display growth-faltering patterns characteristic of resource poor countries, as shown by evidence of cell-mediated enteropathy across a range of nutritional states (Campbell et al. 2003). Despite focused nutrition interventions (e.g., zinc and iron supplementation), growth faltering continues, prompting the need to investigate other EED causal factors.

## Exposome Perspectives for EED and Stunting

Children susceptible to undernutrition suffer from a vicious cycle of diseases, malnutrition, and stunting in low- and middle-income countries (Keusch et al. 2013). Given the effects of the microbiome and microbiota on growth and development, there is need to determine the effect of both environmental chemicals on microbes and microbe modifications to environmental chemicals. Our knowledge of the metabolome should be integrated with the gut microbiota of children, both healthy and stunted, in EED-endemic regions to determine the relationship between host-, microbe-, and diet-derived metabolites and EED (Brown et al. 2015; Ray 2015; Smith et al. 2013; Subramanian et al. 2014). Examining the combined effect of environmental exposures and nutrition in EED animal models and children with EED may also give insights into the basic characteristics of an EED microbiome. Improved understanding of the impact of a child’s early-life exposome on health is important in the diagnosis, treatment, and prevention of EED (Caricilli et al. 2014). Investigation of the human microbiome, proteome, and metabolome opens up new frontiers for EED research, and these approaches should be fully used to characterize exposure biomarkers in infancy and childhood. Many studies attempt to show the role of microbes or microbial effects on EED development (Crane et al. 2015; Keusch et al. 2013), further stimulating discussions about the responsible causal agents. The gut microbiota varies widely among individuals and geographical locations; this may be attributable to exposome variation in dietary patterns, pathogens, pesticides, drugs, and environmental pollutants. Use of microbial population genetics and metabolomics to describe EED phenotypes across regions is a promising integrative systems biology approach to help discover unknown causes of EED with respect to environmental exposures. For instance, gnotobiotic mice have been used to test the effect of diet, environmental chemicals and toxicants on the gut microbiota and host gastrointestinal physiology (Subramanian et al. 2014; Turnbaugh et al. 2009).

The chronic nature of EED can exacerbate persistent malnutrition and micronutrient deficiencies; hence, there is need to search for new targets for nutritional interventions (McKay et al. 2010). Children in the first 2 years of life have a high likelihood of stunting because of suboptimal breast and complementary feeding practices, micronutrient deficiencies (Barker 2007), poor sanitation and recurrent infections, exposures from mycotoxins, heavy metals, and chemical pollutants (Prendergast and Humphrey 2014) as well as exposure to organic pesticides, as demonstrated by the presence of their residues in breast milk (Mishra and Sharma 2011; Zhou et al. 2011). The child’s ability to reverse the risk of stunting may be further reduced if intervention does not take place in the first 2 years of life, a critical period (Barker 2007), especially if the environment remains resource constrained or food insecure (Martorell and Zongrone 2012; Prendergast and Humphrey 2014; Victora et al. 2010). This critical window was demonstrated in a study of healthy Zimbabwe infants where critical periods of poor linear growth were associated with low-grade chronic inflammation (Prendergast et al. 2014). In addition, specific pathogens or chemical toxicants may aggravate stunting via chronic infections, inflammation, and or gut mucosal damage (Jones et al. 2014).

**Figure 2:**
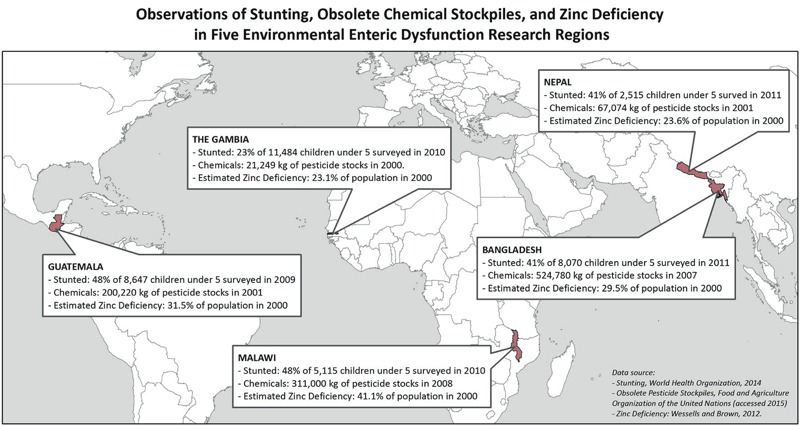
Observations of stunting, obsolete chemical stockpiles, and zinc deficiency in five environmental enteric dysfunction research regions: Guatemala, Malawi, The Gambia, Bangladesh, and Nepal. This simplified GIS map shows two variables from the exposome—chemical pesticides and zinc deficiency—and associations in known EED-affected countries alongside stunting. Data source for stunting: World Health Organization (2014). Data source for obsolete chemical stockpiles: Food and Agriculture Organization of the United Nations (2015). Data source for zinc deficiency: Wessells and Brown (2012).

Nutritional rehabilitation of severely or moderately malnourished children in low-income countries should be accompanied by a clear understanding of the impact of diet and environmental toxicants on the overall nutrition profile and growth and development parameters. There are limited data about the adverse health effects associated with exposure to multiple environmental sources, so there is need to examine the toxicological effects on the developing GI mucosa and immune systems resulting from cumulative exposures to environmental contaminants (Nweke and Sanders 2009). Geographic information systems (GIS) mapping may be a useful tool in examining the spatial distribution of the exposome in conjunction with other measures. Specifically, EED has been observed alongside stunting in The Gambia, Guatemala, Bangladesh, and Malawi (Crane et al. 2015). Using these selected EED research regions, we created a simplified GIS map (Figure 2) to demonstrate how variables such as stunting, chemical exposures, and zinc deficiency illustrate the rationale for further investigation of an EED exposome. Given that the prevalence of low height-for-age (stunting) in children < 5 years old has been recommended as an indirect indicator of a population’s risk for zinc deficiency (Wessells and Brown 2012), additional exposures and exposome factors could also be geospatially mapped in this context. Correlations between the exposome and EED may lead to novel insights of high relevance to environmental health. With a better understanding of the biomarkers of chemical exposure and EED, we envision a future scenario under which EED can be prevented and treated using multiple approaches that will include a clean living environment, hygiene, better nutrition, and perhaps restored, mature homeostatic gut microbial ecosystems that do not result in growth stunting.

## Conclusions and Future Research Needs

The exposome includes a broad spectrum of possible factors that may be involved in EED. This concept offers a novel perspective for chemical toxicants’ roles in disrupting microbiota maturity, because chemical toxicants are embedded in the microbial ecosystem and affect how the gut and immune system develop in the first few years of life. Chemical exposures may play a crucial role in early-childhood growth and development via multiple mechanisms, and merit inclusion in EED studies across geographically diverse regions. Integration of serum metabolomics and proteomics could reveal an EED-associated exposome across developing nations that could lead to novel and promising approaches to identify, validate, and differentiate EED globally. Mechanistic connections among chemical toxicant exposures, immunity, and the gut microbiota ecosystem during growth and development may guide future EED therapeutic studies to alleviate stunting. A compelling reason for embracing the exposome in EED is the potential for bi-directional relationships to emerge between biomarkers of exposure and biomarkers of disease, as well as to identify innovative combinations of preventive and therapeutic approaches that can sustainably reduce EED prevalence globally.

## References

[r1] Agapova S, Stephenson K, Manary M, Weisz A, Tarr PI, Mkakosya R (2013). Detection of low-concentration host mRNA transcripts in Malawian children at risk for environmental enteropathy.. J Pediatr Gastroenterol Nutr.

[r2] Barker DJP (2007). Introduction: the window of opportunity.. J Nutr.

[r3] Bartelt LA Lima AA Kosek M Peñataro Yori P Lee G Guerrant RL 2013 “Barriers” to child development and human potential: the case for including the “neglected enteric protozoa” (NEP) and other enteropathy-associated pathogens in the NTDs. PLoS Negl Trop Dis 7 e2125, doi:10.1371/journal.pntd.0002125 23593514 PMC3623703

[r4] Breton J Massart S Vandamme P De Brandt E Pot B Foligné B 2013 Ecotoxicology inside the gut: impact of heavy metals on the mouse microbiome. BMC Pharmacol Toxicol 14 62, doi:10.1186/2050-6511-14-62 24325943 PMC3874687

[r5] Brown EM Wlodarska M Willing BP Vonaesch P Han J Reynolds LA 2015 Diet and specific microbial exposure trigger features of environmental enteropathy in a novel murine model. Nat Commun 6 7806, doi:10.1038/ncomms8806 26241678 PMC4532793

[r6] Campbell DI, McPhail G, Lunn PG, Elia M, Jeffries DJ (2004). Intestinal inflammation measured by fecal neopterin in Gambian children with enteropathy: association with growth failure, *Giardia lamblia*, and intestinal permeability.. J Pediatr Gastroenterol Nutr.

[r7] Campbell DI, Murch SH, Elia M, Sullivan PB, Sanyang MS, Jobarteh B (2003). Chronic T cell-mediated enteropathy in rural west African children: relationship with nutritional status and small bowel function.. Pediatr Res.

[r8] Caricilli AM, Castoldi A, Câmara NO (2014). Intestinal barrier: a gentlemen’s agreement between microbiota and immunity.. World J Gastrointest Pathophysiol.

[r9] Crane RJ, Jones KD, Berkley JA (2015). Environmental enteric dysfunction: an overview.. Food Nutr Bull.

[r10] Daniell E, Ryan EP (2012). The nutrigenome and gut microbiome: chronic disease prevention with crop phytochemical diversity.. In: The Molecular Basis of Plant Genetic Diversity (Caliskan M, ed).

[r11] De Filippo C, Cavalieri D, Di Paola M, Ramazzotti M, Poullet JB, Massart S (2010). Impact of diet in shaping gut microbiota revealed by a comparative study in children from Europe and rural Africa.. Proc Natl Acad Sci USA.

[r12] de Onis M, Dewey KG, Borghi E, Onyango AW, Blössner M, Daelmans B (2013). The World Health Organization’s global target for reducing childhood stunting by 2025: rationale and proposed actions.. Matern Child Nutr.

[r13] Dorrestein PC, Mazmanian SK, Knight R (2014). Finding the missing links among metabolites, microbes, and the host.. Immunity.

[r14] dos Reis JC, de Morais MB, Oliva CA, Fagundes-Neto U (2007). Breath hydrogen test in the diagnosis of environmental enteropathy in children living in an urban slum.. Dig Dis Sci.

[r15] Food and Agriculture Organization of the United Nations (2015). Prevention and Disposal of Obsoloete Pesticides.. http://www.fao.org/agriculture/crops/obsolete-pesticides/where-stocks/africa-stocks/en/.

[r16] Galpin L, Manary MJ, Fleming K, Ou CN, Ashorn P, Shulman RJ (2005). Effect of *Lactobacillus* GG on intestinal integrity in Malawian children at risk of tropical enteropathy.. Am J Clin Nutr.

[r17] Gordon JI Dewey KG Mills DA Medzhitov RM 2012 The human gut microbiota and undernutrition. Sci Transl Med 4 137ps112, doi:10.1126/scitranslmed.3004347 22674549

[r18] Hall AM, Ward FJ, Shen CR, Rowe C, Bowie L, Devine A (2007). Deletion of the dominant autoantigen in NZB mice with autoimmune hemolytic anemia: effects on autoantibody and T-helper responses.. Blood.

[r19] Humphrey JH (2009). Child undernutrition, tropical enteropathy, toilets, and handwashing.. Lancet.

[r20] Jones KD, Thitiri J, Ngari M, Berkley JA (2014). Childhood malnutrition: toward an understanding of infections, inflammation, and antimicrobials.. Food Nutr Bull.

[r21] Kau AL, Ahern PP, Griffin NW, Goodman AL, Gordon JI (2011). Human nutrition, the gut microbiome and the immune system.. Nature.

[r22] Kelly P, Menzies I, Crane R, Zulu I, Nickols C, Feakins R (2004). Responses of small intestinal architecture and function over time to environmental factors in a tropical population.. Am J Trop Med Hyg.

[r23] Keusch GT, Denno DM, Black RE, Duggan C, Guerrant RL, Lavery JV (2014). Environmental enteric dysfunction: pathogenesis, diagnosis, and clinical consequences.. Clin Infect Dis.

[r24] Keusch GT, Rosenberg IH, Denno DM, Duggan C, Guerrant RL, Lavery JV (2013). Implications of acquired environmental enteric dysfunction for growth and stunting in infants and children living in low- and middle-income countries.. Food Nutr Bull.

[r25] Koenig JE, Spor A, Scalfone N, Fricker AD, Stombaugh J, Knight R (2011). Succession of microbial consortia in the developing infant gut microbiome.. Proc Natl Acad Sci USA.

[r26] Langford R, Lunn P, Panter-Brick C (2011). Hand-washing, subclinical infections, and growth: a longitudinal evaluation of an intervention in Nepali slums.. Am J Hum Biol.

[r27] Lin A, Arnold BF, Afreen S, Goto R, Huda TM, Haque R (2013). Household environmental conditions are associated with enteropathy and impaired growth in rural Bangladesh.. Am J Trop Med Hyg.

[r28] Lindenbaum J (1973). Tropical enteropathy.. Gastroenterology.

[r29] Lindenbaum J, Harmon JW, Gerson CD (1972). Subclinical malabsorption in developing countries.. Am J Clin Nutr.

[r30] Lindenmayer GW, Stoltzfus RJ, Prendergast AJ (2014). Interactions between zinc deficiency and environmental enteropathy in developing countries.. Adv Nutr.

[r31] Lioy PJ Rappaport SM 2011 Exposure science and the exposome: an opportunity for coherence in the environmental health sciences [Editorial]. Environ Health Perspect 119 A466 A467, doi:10.1289/ehp.1104387 22171373 PMC3226514

[r32] Martorell R, Zongrone A (2012). Intergenerational influences on child growth and undernutrition.. Paediatr Perinat Epidemiol.

[r33] Maurice CF, Haiser HJ, Turnbaugh PJ (2013). Xenobiotics shape the physiology and gene expression of the active human gut microbiome.. Cell.

[r34] Mbuya MN Humphrey JH 2016 Preventing environmental enteric dysfunction through improved water, sanitation and hygiene: an opportunity for stunting reduction in developing countries. Matern Child Nutr 12(suppl 1) 106 120, doi:10.1111/mcn.12220 26542185 PMC5019251

[r35] McKay S, Gaudier E, Campbell DI, Prentice AM, Albers R (2010). Environmental enteropathy: new targets for nutritional interventions.. Int Health.

[r36] Mduma ER, Gratz J, Patil C, Matson K, Dakay M, Liu S (2014). The Etiology, Risk Factors and Interactions of Enteric Infections and Malnutrition and the Consequences for Child Health and Development (MAL-ED): description of the Tanzanian site.. Clin Infect Dis.

[r37] Miller GW, Jones DP (2014). The nature of nurture: refining the definition of the exposome.. Toxicol Sci.

[r38] Mishra K, Sharma RC (2011). Assessment of organochlorine pesticides in human milk and risk exposure to infants from North-East India.. Sci Total Environ.

[r39] Ngure FM, Reid BM, Humphrey JH, Mbuya MN, Pelto G, Stoltzfus RJ (2014). Water, sanitation, and hygiene (WASH), environmental enteropathy, nutrition, and early child development: making the links.. Ann N Y Acad Sci.

[r40] Nweke OC Sanders WH III 2009 Modern environmental health hazards: a public health issue of increasing significance in Africa. Environ Health Perspect 117 863 870, doi:10.1289/ehp.0800126 19590675 PMC2702398

[r41] Pop M Walker AW Paulson J Lindsay B Antonio M Hossain MA 2014 Diarrhea in young children from low-income countries leads to large-scale alterations in intestinal microbiota composition. Genome Biol 15 R76, doi:10.1186/gb-2014-15-6-r76 24995464 PMC4072981

[r42] Prendergast AJ, Humphrey JH (2014). The stunting syndrome in developing countries.. Paediatr Int Child Health.

[r43] Prendergast AJ, Humphrey JH, Mutasa K, Majo FD, Rukobo S, Govha M (2015). Assessment of environmental enteric dysfunction in the SHINE trial: methods and challenges.. Clin Infect Dis.

[r44] Prendergast A, Kelly P (2012). Enteropathies in the developing world: neglected effects on global health.. Am J Trop Med Hyg.

[r45] Prendergast AJ Rukobo S Chasekwa B Mutasa K Ntozini R Mbuya MN 2014 Stunting is characterized by chronic inflammation in Zimbabwean infants. PloS One 9 e86928, doi:10.1371/journal.pone.0086928 24558364 PMC3928146

[r46] Rappaport SM (2011). Implications of the exposome for exposure science.. J Expo Sci Environ Epidemiol.

[r47] Rappaport SM (2012). Biomarkers intersect with the exposome.. Biomarkers.

[r48] Rappaport SM Barupal DK Wishart D Vineis P Scalbert A 2014 The blood exposome and its role in discovering causes of disease. Environ Health Perspect 122 769 774, doi:10.1289/ehp.1308015 24659601 PMC4123034

[r49] Rappaport SM, Smith MT (2010). Epidemiology. Environment and disease risks.. Science.

[r50] Ray K 2015 Malnutrition: new mouse model of EE. Nat Rev Gastroenterol Hepatol 12 489, doi:10.1038/nrgastro.2015.144 26284563

[r51] Salazar-Lindo E, Allen S, Brewster DR, Elliott EJ, Fasano A, Phillips AD (2004). Intestinal infections and environmental enteropathy: Working Group report of the second World Congress of Pediatric Gastroenterology, Hepatology, and Nutrition.. J Pediatr Gastroenterol Nutr.

[r52] Shen L, Turner JR (2006). Role of epithelial cells in initiation and propagation of intestinal inflammation. Eliminating the static: tight junction dynamics exposed.. Am J Physiol Gastrointest Liver Physiol.

[r53] Smith LE, Stoltzfus RJ, Prendergast A (2012). Food chain mycotoxin exposure, gut health, and impaired growth: a conceptual framework.. Adv Nutr.

[r54] Smith MI, Yatsunenko T, Manary MJ, Trehan I, Mkakosya R, Cheng J (2013). Gut microbiomes of Malawian twin pairs discordant for kwashiorkor.. Science.

[r55] Subramanian S, Huq S, Yatsunenko T, Haque R, Mahfuz M, Alam MA (2014). Persistent gut microbiota immaturity in malnourished Bangladeshi children.. Nature.

[r56] Turnbaugh PJ Ridaura VK Faith JJ Rey FE Knight R Gordon JI 2009 The effect of diet on the human gut microbiome: a metagenomic analysis in humanized gnotobiotic mice. Sci Transl Med 1 6ra14, doi:10.1126/scitranslmed.3000322 PMC2894525 20368178

[r57] Victora CG, de Onis M, Hallal PC, Blössner M, Shrimpton R (2010). Worldwide timing of growth faltering: revisiting implications for interventions.. Pediatrics.

[r58] Vrijheid M Slama R Robinson O Chatzi L Coen M van den Hazel P 2014 The Human Early-Life Exposome (HELIX): project rationale and design. Environ Health Perspect 122 535 544, doi:10.1289/ehp.1307204 24610234 PMC4048258

[r59] Wessells KR Brown KH 2012 Estimating the global prevalence of zinc deficiency: results based on zinc availability in national food supplies and the prevalence of stunting. PloS One 7 e50568, doi:10.1371/journal.pone.0050568 23209782 PMC3510072

[r60] World Health Organization (2014). Nutrition: Global Targets Tracking Tool.. http://www.who.int/nutrition/trackingtool/en/.

[r61] Wu GD, Chen J, Hoffmann C, Bittinger K, Chen YY, Keilbaugh SA (2011). Linking long-term dietary patterns with gut microbial enterotypes.. Science.

[r62] Yatsunenko T, Rey FE, Manary MJ, Trehan I, Dominguez-Bello MG, Contreras M (2012). Human gut microbiome viewed across age and geography.. Nature.

[r63] Zhang L Nichols RG Correll J Murray IA Tanaka N Smith PB 2015 Persistent organic pollutants modify gut microbiota–host metabolic homeostasis in mice through aryl hydrocarbon receptor activation. Environ Health Perspect 123 679 688, doi:10.1289/ehp.1409055 25768209 PMC4492271

[r64] Zhou P, Wu Y, Yin S, Li J, Zhao Y, Zhang L (2011). National survey of the levels of persistent organochlorine pesticides in the breast milk of mothers in China.. Environ Pollut.

